# Summer day-roost selection by eastern red bats varies between areas with different land-use histories

**DOI:** 10.1371/journal.pone.0237103

**Published:** 2020-08-24

**Authors:** Maria N. Monarchino, Marnie L. Behan, Joseph S. Johnson

**Affiliations:** 1 Department of Biological Sciences, Ohio University, Athens, Ohio, United States of America; 2 School of Environment and Natural Resources, The Ohio State University, Athens, Ohio, United States of America; University of Western Ontario, CANADA

## Abstract

The eastern red bat (*Lasiurus borealis*) is widely considered to be in decline, inspiring interest in identifying important habitats for conservation in the eastern United States. Unfortunately, knowledge of important day-roosting habitats is lacking for much of the species’ range. We examined patterns of day-roost selection by male and female eastern red bats at two study sites in southeastern Ohio, U. S. A, to help fill this information gap. We radio-tagged 28 male and 25 female bats during the summers of 2016–2019 and located 53 male and 74 female roosts. Day-roost selection differed between sexes and study areas. In a mostly even-aged forest with significant historical disturbance, we found males and females roosting in trees located at higher elevations, with no clear selection based on tree or stand characteristics. Specifically, males selected trees with larger diameters located at lower, cooler elevations than females, which selected smaller diameter trees found at higher, warmer elevations. However, in a forest with less historical disturbance and more structural diversity, we found sexes differed in how they selected from available habitats. These data show that heterogeneity in environmental conditions can lead to different patterns in selection, even between sites located within a small geographic area. They also show that eastern red bats sexually segregate on the local landscape in the presence of diverse forest conditions but may not do so in the absence of such diversity. We recommend managing forests to maintain structural diversity across an elevational gradient to provide male and female eastern red bats with suitable day-roosting habitat in southeast Ohio.

## Introduction

Successful wildlife conservation relies on accurate descriptions of the habitat features and resources that species need to survive and reproduce [1]. However, such descriptions may be difficult to formulate for species with large geographic ranges, as they may occupy a wide range of environmental conditions and ecological communities [2, 3]. Further hindering identification of important resources, some wide-ranging species also occupy human-altered landscapes composed of a mosaic of habitat patches, each with a distinct vegetative composition and structure that influences habitat use and quality [4, 5]. Although this can be a challenge for conservation, these patches represent opportunities to study how species utilize or fail to utilize different habitats within the same region, which may help improve management. Additionally, as the number of factors threating biodiversity continues to increase, improving habitat for wildlife will remain a priority for conservation [6].

In North America, the eastern red bat (*Lasiurus borealis*) is a wide-ranging species believed to be in decline as a result of collisions with commercial wind turbines and habitat loss [7, 8]. Several species of bats suffer from fatal collisions at commercial wind-energy facilities, but the eastern red bat is one of the three most commonly killed species in the Midwest [9, 10]. Exact mortality rates are unclear and vary by year, but an estimated 143,023 eastern red bats were killed at wind facilities in the United States from 2000 to 2011 [10]. Eastern red bat mortalities are expected to continue as wind-energy technology becomes more popular and efficient, leading to concerns over their population viability. While large-scale mortalities at wind-energy facilities are a newer phenomenon, it has long been assumed that eastern red bats are negatively affected by loss of forest habitat across their range [11, 12]. As a species that completely relies on forests, red bats are responsive to changes in forest communities, and forest management conducted with this bat in mind may help slow its decline [13]. Unfortunately, studies of habitat selection in this species are sparsely distributed across its range, which includes the entirety of the eastern United States and Canada, as well as parts of Mexico [14].

Forests are central to the ecology of many bat species, with 27 of the 45 species documented in the United States and Canada known to roost in trees [15]. Day-roosts are important because they provide bats with shelter from the elements, protection from predators, and a place to raise young [12, 16]. Selection of day-roosts are known to vary widely both within and among species. Within species, males and females often select different summer roosts based on their differing needs and limitations during this time [17, 18]. In contrast to males, which often select cooler roosting environments to facilitate torpor during summer [19, 20], females require warm roosts to help keep thermoregulatory costs as low as possible during gestation and lactation [21, 22]. In species that roost under bark or in tree cavities, males tend to roost more solitarily while females often select large trees that have space for social groups that significantly increase roost temperatures and provide energy savings [21–23]. While it is common to see females exhibiting different roosting strategies from males, such as social roosting, this may vary among species. Solitary, foliage roosting species, such as the eastern red bat, which hangs from branches and leaf petioles, lack the benefits of social roosting in both sexes [14]. Because these bats roost in tree canopies exposed to weather, eastern red bats may select roosts based on temperature to help compensate for thermally unstable roosting environments [24]. However, there are few studies that examine the role of temperature and other habitat characteristics in roosting by red bats, especially considering the large geographic range of this species.

Several characteristics of day-roosts influence roost temperature. Foliage roosting species have been found selecting roosts that are taller than the height of the surrounding canopy, which may increase solar exposure and roost temperature [25, 26]. Other foliage species such as the hoary bat (*Lasiurus cinereus*) choose roost sites that decrease wind exposure, offsetting convective heat loss [27]. Red bats are also known to select roosts based on characteristics that have little or no known connection to temperature. For example, red bats have been found roosting high in mature forest canopies that provide adequate gaps beneath the roost for quick entry and exit but enough cover to avoid detection by site-oriented predators [12, 28]. Additionally, most studies on eastern red bats have found this species to have an affinity for large diameter hardwoods [29–31]. However, it is unclear if this roost characteristic is connected to temperature or some other benefit for foliage roosting bats. Thus, day-roost selection in bats is influenced by several variables pertaining to habitat at different spatial scales.

As a result of red bats being influenced by forest structure and composition, forest-management regimes are likely to affect habitat selection for this species. For example, in contiguous forests in Kentucky, red bats roost in mature hardwood stands with high basal area far from forest edges [31]. In intensively managed forests in the southern United States, these bats roost within or close to mature hardwood stands with a closed canopy [26] or in pine stands that retained mature hardwoods [32]. While these studies provide insight as to how bats utilize managed forests, many forests are not intensively managed, but are left to regenerate following disturbance. This results in different forest structures and possibly different patterns of habitat use. This is true of many forests in the Western Allegheny Plateau Ecoregion [33], where not only have forests been heavily disturbed and remain unmanaged, but also where there is currently no local information on roosting behaviors of the eastern red bat. Determining habitat characteristics important to eastern red bats in this region may help local management agencies to promote or protect habitat for this declining bat species.

The purpose of this study was to compare day-roost selection by male eastern red bats to that of females in southeastern Ohio forests with markedly different land use histories and forest structures. We used an information theoretic approach to rank a suite of *a priori* models seeking to explain day-roost selection within each site. This approach allowed for a comparison of top models of selection in each study location to determine if patterns in selection differ. We hypothesized that (i) eastern red bats would select day-roosts non-randomly, with different habitat characteristics used by males and females, and (ii) that selection patterns would differ between sites with different forest conditions.

## Materials and methods

Approval for research was given by the Institutional Animal Care and Use Committee of Ohio University (17-H-008) and the necessary permits (Endangered Species Recovery Permit # TE5674B) were acquired before conducting field work. All study methods followed the guidelines of the American Society of Mammalogists [34].

### Study areas

Our study was conducted at two locations in Hocking County, Ohio, each with different land use histories and forest communities. Our first site, Crane Hollow Nature Preserve (N 39.480089, W -82.584173), is approximately 768 hectares of state nature preserve. Crane Hollow Preserve is a biologically diverse area with ≥10,000 documented floral and faunal species inhabiting the ridgetops and Blackhand Sandstone gorges that run the length of the preserve. Elevation at this location ranges from 335 m on ridges to 228 m at the bottom of the gorges. The forest community along ridgetops is dominated by oak (*Quercus* spp.), maple (*Acer* spp.), hickory (*Carya* spp.), and elm species (*Ulmus* spp.), as well as American beech (*Fagus grandifolia*) and tulip poplar (*Liriodendron tulipifera*). Eastern hemlock (*Tsuga canadensis*), American sycamore (*Platanus occidentalis*), American beech, birch (*Betula* sp.), tulip poplar, and maple species dominate the steep slopes and bottoms of the gorges. Although extensive farming and timber harvesting has occurred across the preserve before the property became a state preserve in 1977, large portions of Crane Hollow Preserve have maintained stands that are estimated to be over 100 years old.

Our second site, located on 1348 hectares owned by the Ohio Division of Wildlife approximately 33 km northeast of Crane Hollow Preserve, is a section of O’Dowd Wildlife Area locally referred to as Sunday Creek Coal (N 39.531083, W -82.198662). From the late 1800’s to the 1950’s, Sunday Creek Coal was the location of large coal mining operations that, along other industry activities, left the property deforested. Much of the land has naturally reforested since the closing of the final mine in the 1950’s, although disturbances such as timber harvests, oil and natural gas exploitation, and the creation of extensive ATV trails are common. The topography at Sunday Creek Coal is characterized by hillsides built up by surface mining. Slopes are not as precipitous as Crane Hollow Preserve and lacked exposed cliffs, but elevations were similar, ranging from 222 to 323 m. The forest community consists of a variety of tree species commonly found in Ohio, such as oaks maple, tulip poplar, bigtooth aspen (*Populus grandidentata*), American beech, American sycamore, and elms species. Due to the recent mining operations, stands at Sunday Creek Coal tended to be more densely stocked with smaller, younger stems compared to those at Crane Hollow Preserve.

### Capture and radio-telemetry

We captured bats from late May through early August 2016–2019 using mist nets (Avinet Inc, Portland, Maine) placed in foraging corridors such as service roads, streams, or over open water sources. We opened nets shortly before sunset and left them open for 5 hours. All captured bats were identified to species, age (adult or young-of-the-year), sex, and reproductive condition. Right forearm length and weight were also measured. Eastern red bats weighing >6.6 g were fitted with 0.33 g transmitters (model LB-2 and LB-2TX, Holohil Systems Ltd., Ontario, Canada) using surgical adhesive (Osto-bond, Quebec, Canada). We tracked radio-tagged bats to their day-roosts using handheld receivers (Biotracker Version, Lotek Wireless, Ontario, Canada) each day for the life of the transmitter or until the transmitter had fallen off. We recorded the UTM of each day-roost using a handheld GPS unit with a 3 m accuracy.

### Day-roosting habitat

To compare habitats used to those available, we collected a suite of measurements at the tree-, stand-, and landscape-scale at each day-roost and at 100 random trees in each study area. Tree-scale habitat measurements included tree species, diameter at breast height (DBH), tree height, percent canopy cover, and slope aspect. Stand-scale measurements included canopy height, distance to nearest live tree, percent slope, and basal area. Landscape-scale measurements included distance to roads, distance to water, distance to forest edge, and elevation. Basal area was measured using a 10-factor wedge prism, height was estimated with a laser rangefinder/hypsometer, percent canopy cover was determined using a spherical densitometer, and DBH was measured using a DBH tape. Landscape-scale measurements were calculated using ArcMap [35].

We generated random locations in each study area to measure habitat available for day-roosting. Before generating these locations, we determined the area covered by dominant habitat types within each study site and dispersed 100 points proportionally within each of those habitats. Dominant habitat types were provided by previous studies [36] and from the Ohio Department of Natural Resources. At Crane Hollow Preserve, these habitats were oak/mesic forests, hemlock forests, and forested floodplains. At Sunday Creek Coal, the dominant habitat types were xeric forests, mesic forests, floodplain forests, and a small amount of unclassified habitat. Coordinates for random points were created using a random point generator in ArcMap. We navigated to these locations using a handheld GPS and collected the same suite of habitat variables described above for a tree >10 cm in diameter located closest to the random point.

### Ambient temperatures

To investigate if day-roost selection is influenced by ambient air temperatures (*T*_*a*_), we measured *T*_*a*_ across the elevational gradient present in each study area. We deployed temperature and relative humidity dataloggers (Onset MX2301, Onset Computer Corp., Bourne, Massachusetts) inside solar radiation shields at three slope positions. These corresponded to ridgetops (Crane Hollow Preserve: 334 m; Sunday Creek Coal: 323 m), mid-slopes (288 and 270 m, respectively), and bottoms (228 and 222 m, respectively). Each logger was programmed to record *T*_*a*_ every ten minutes. Dataloggers were deployed in wooded areas and affixed to small diameter trees approximately 1 m from the ground. Because we were interested in how temperatures varied across slope positions during the day-roosting period, we summarized *T*_*a*_ recorded at each logger during the hours between sunrise and sunset into daily average temperatures. In addition, we further limited our summary to data collected during the summer (June–August) of 2018.

### Data analysis

Male and female eastern red bat capture rates were calculated for each site by dividing the total number of each sex captured by capture effort (nights of netting) to provide a measure of capture success. We did not statistically compare capture rates as mist-netting is not a reliable technique for measuring abundance [37], but we do present capture rates as a qualitative measure of capture success. To determine if forest conditions differed between our two study areas, we conducted a two-sample t-test on forest measures such as basal area, DBH, tree height, and number of stems within 20 m from our random trees at both sites. In addition, we conducted a two-sided Fishers exact test of independence between our female, male, and available trees at both sites to determine if bats were using certain species more than expected. Similarly, we used a Fisher’s exact test to determine whether available tree species differed between the two study locations.

To test our two hypotheses, we compared the habitat characteristics of male roosts, female roosts, and random trees at each site using multinomial logistic regression and a model selection approach. We used roost trees as the experimental unit and assumed all trees to be independent. To assess if using trees, for which we gathered an unequal sample size among bats, as the experimental unit biased our analyses, we generated average roost characteristics for each bat and compared the distribution of these average values to that of all trees (data not shown). These distributions were similar, and we used individual trees in our analysis. Prior to analysis, we tested for multicollinearity in RStudio [38] using a correlation matrix and did not include variables in a model if they were > 70% correlated. We ran 20 multinomial logistic regression models for each study area, with each model representing a distinct, biologically informed *a priori* model developed from a review of existing literature (S1 Table). Our dependent variable was sex, which was organized into three categories (male, female, and random trees) to compare roosts of each sex to available habitat. To assess our hypothesis that day-roost selection would differ between the two areas, we ranked models for each study area separately using Akaike’s Information Criterion corrected for small sample sizes (AICc). Models with ΔAICc ≤ 2 were considered competitive as a top model [39]. Using this model selection approach, we compared our top models from each study location to assess our hypothesis that habitat selection would differ between the two study areas. We assessed the fit of our top models using Hosmer-Lemeshow goodness of fit tests and referenced our odds ratios to see which variables were driving our top models. Confidence ratios for our odds ratios were also assessed and any variable with confidence intervals crossing 1 were not considered to be a strong predictor.

## Results

### Capture and radio-telemetry

We captured bats on 69 nights, including 39 nights at Crane Hollow Preserve and 30 nights at Sunday Creek Coal. Adult males were more commonly captured than adult females at both sites. We captured 1.1 adult male and 0.6 adult female red bats/night at Crane Hollow Preserve (*n* = 43 male, 22 female), and 0.9 adult male and 0.2 adult female bats/night captured at Sunday Creek Coal (*n* = 28 male, 7 female). We radio-tagged 17 adult females and 14 adult males at Crane Hollow Preserve, tracking these bats to 89 roosts (male: *n* = 29, female: *n* = 60). At Sunday Creek Coal, we radio-tagged 7 adult females and 16 adult males, tracking them to 51 roosts (male: *n* = 34, female: *n* = 17). Overall, bats were tracked for an average of 6.5 days (range = 1–19) to an average of 3.3 day-roosts (range = 1–11) per bat. Bats were captured in the highest density during the months of June and July at both of our study locations. Males were most captured during June (n = 38) and July (n = 40), while females had the highest numbers of captures in June (n = 14). Pregnant bats were detected throughout May (n = 5) and June (n = 7), while lactating bats appeared (n = 4) in June and continued into July (n = 5). Post-lactating females (n = 4) were detected from mid- to late-July.

### Day-roosting and available habitat

Eastern red bats switched roosts frequently at both study areas. On average, bats switched roost trees every 1.3 days (range = 1–3) at Sunday Creek Coal and every 1.1 days (range = 1–14) at Crane Hollow Preserve. At Sunday Creek Coal, females switched every 1.0 days and males switched every 1.1 days (range = 1–3). Female bats at Crane Hollow Preserve switched roosts every 1.4 days (range = 1–14) and males switched every 1.1 days (range = 1–3). Habitat measurements from our random trees indicated a significantly different forest structure and composition between the study areas. Mean basal area at Sunday Creek Coal was 16.3 ± 0.86 m^2^/ha compared to 23.0 ± 1.1 m^2^/ha at Crane Hollow Preserve (t(189) = 4.87, p > 0.001). Tree diameter averaged 30.4 ± 1.2 cm at Sunday Creek Coal compared to 37.8 ± 5.2 cm at Crane Hollow Preserve (t(184) = 3.84, p < 0.001), and average tree height was 18.7 ± 0.47 m at Sunday Creek Coal compared to 20.5 ± 0.67 cm (t(178) = 2.25, p = 0.03). Finally, number of live stems within 20 m at Sunday Creek Coal was 69.8 ± 4.6 compared to 51.9 ± 3.0 stems at Crane Hollow Preserve (t(195) = -4.74, p < 0.001).

The species composition of the 100 random trees sampled at each site reflect different forest communities (p < 0.001). At Crane Hollow Preserve, there was a significant difference between roost tree and available tree species on the landscape (p <0.001). The most frequently used species were sugar maple (n = 29), oak species (n = 20), and tulip poplar (n = 14). Inspection of our residual values shows that for females, sugar maple was used significantly more than expected (Table 1). At Sunday Creek Coal, the residuals also suggest a trend towards greater use of sugar maple and sweet birch than expected for females, but the omnibus tests did not find a significant difference between roost and available trees on the landscape (p = 0.4163). The most frequently used tree species were tulip poplar (n = 13), oak species (n = 10), sugar maple (n = 8), and American beech (n = 8).

**Table 1:** Standardized Pearson’s residuals comparing roost tree species used by male, female, and available trees on the landscape in two study areas in Ohio from 2016–2019.

Species	Female	Male	Available
**Crane Hollow Preserve**			
*Acer rubrum*	-1.592	-0.282	1.385
*Acer saccharum*	**2.417**	-0.289	-1.716
*Betula alleghaniensis*	0.458	1.251	-1.029
*Betula lenta*	-0.656	1.125	-0.098
*Carya spp*.	0.794	-0.959	-0.098
*Fagus grandifolia*	-0.656	-0.959	1.025
*Fraxinus spp*.	0.049	0.795	-0.466
*Juglans nigra*	1.713	-0.554	-1.029
*Liriodendron tulipifera*	-0.088	1.007	-0.474
*Oxydendrum arboretum*	-0.563	-0.392	0.647
*Pinus virginiana*	-1.127	-0.783	1.295
*Platanus occidentalis*	0.049	-0.678	0.328
*Prunus serotine*	-0.797	-0.554	0.916
*Quercus spp*.	0.578	1.394	-1.198
*Robinia pseudoacacia*	-0.563	-0.392	0.647
*Sassafras albidum*	1.211	-0.392	-0.727
*Tsuga canadensis*	**-2.52**	-1.752	**2.895**
*Ulmus spp*.	0.522	0.893	-0.885
**Sunday Creek Coal**			
*Acer rubrum*	0.007	-1.41	0.807
*Acer saccharinum*	-0.332	-0.47	0.405
*Acer saccharum*	**2.004**	-0.583	-0.479
*Ailanthus altissima*	-0.332	-0.47	0.405
*Betula lenta*	**2.678**	-0.47	-0.818
*Carya spp*.	0.415	-0.282	-0.006
*Fagus grandifolia*	-0.7	1.518	-0.588
*Fraxinus spp*.	-0.47	-0.664	0.573
*Liquidambar styraciflua*	-0.332	-0.47	0.405
*Liriodendron tulipifera*	1.086	0.329	-0.63
*Nyssa sylvatica*	0.84	0.124	-0.413
*Ostrya virginiana*	1.162	0.415	-0.711
*Pinus virginiana*	-0.332	-0.470	0.405
*Platanus occidentalis*	-0.94	-1.329	1.145
*Populus grandidentata*	-0.814	0.587	-0.006
*Prunus serotine*	-0.664	-0.94	0.81
*Quercus spp*.	-1.27	0.923	-0.014
*Robinia pseudoacacia*	-0.575	0.415	-0.005
*Ulmus spp*.	0.743	0.853	-0.188

Values ≤ -2 and ≥ 2 are considered influential and are in bold. Positive values indicate greater influence than expected and negative values indicate less influence than expected.

**Fig 1:**
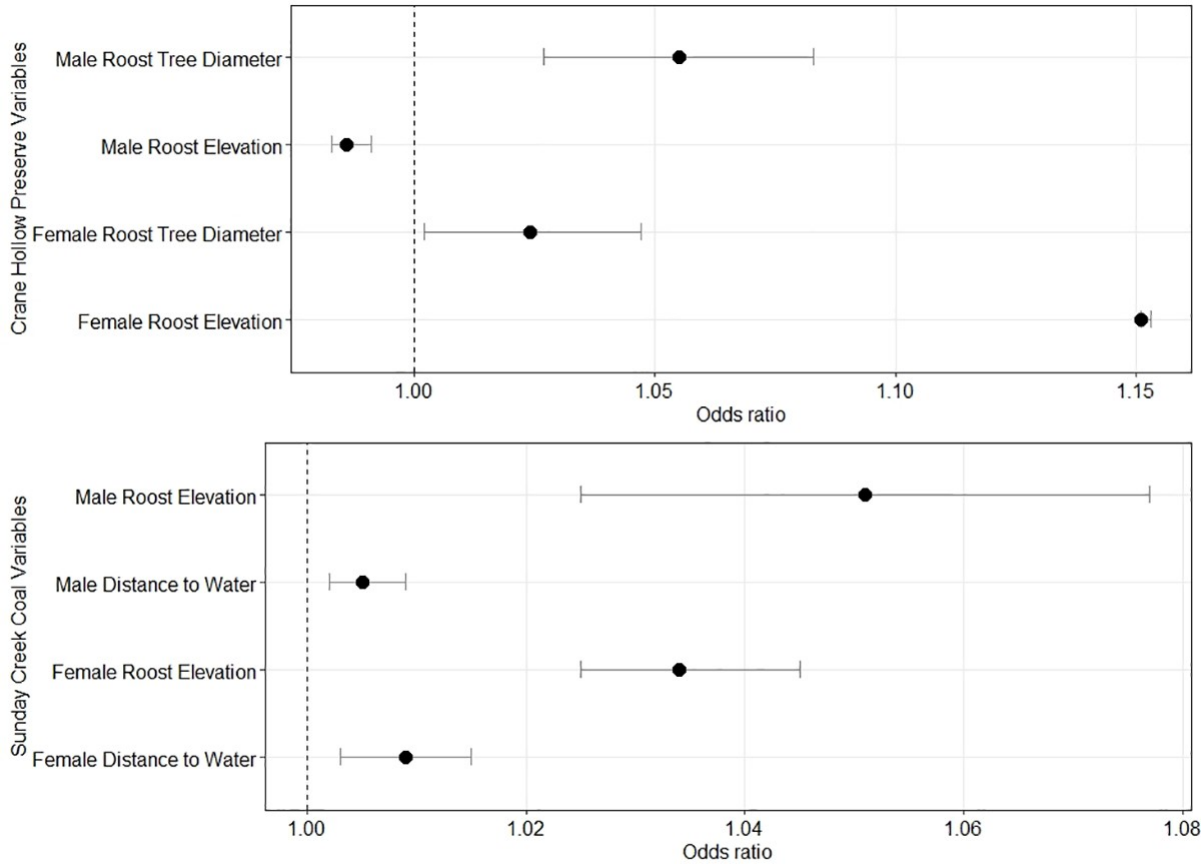
Forest plot representing the odds ratios for each variable included in top models for male and female eastern red bats at Crane Hollow Preserve (top panel) and Sunday Creek Coal (bottom panel) study areas in southeast Ohio.

**Fig 2:**
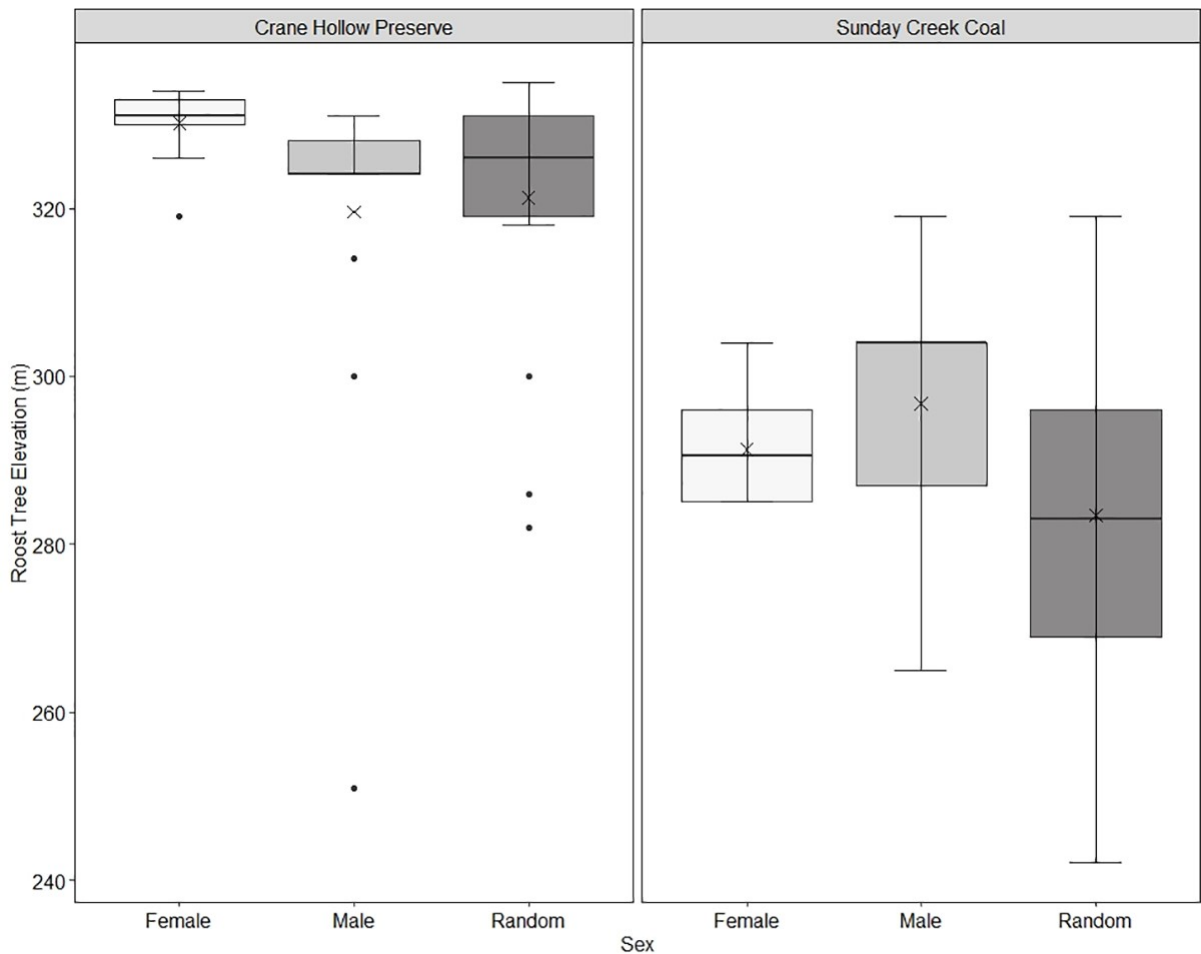
Box and whisker plot showing male (light grey) and female (white) eastern red bats roosting at different elevations than randomly sampled trees (dark grey) at Crane Hollow Preserve and Sunday Creek Coal study areas in southeast Ohio, 2016–2019. Females roosted at higher elevations than males at the preserve, but sexes roosted at similar elevations at the reforested mine. Medians are depicted by the solid line, means are represented by the X’s, and outliers are depicted as black dots. Quartiles 2 and 3 are represented by the box and quartiles 1 and 4 are represented by whiskers.

**Fig 3:**
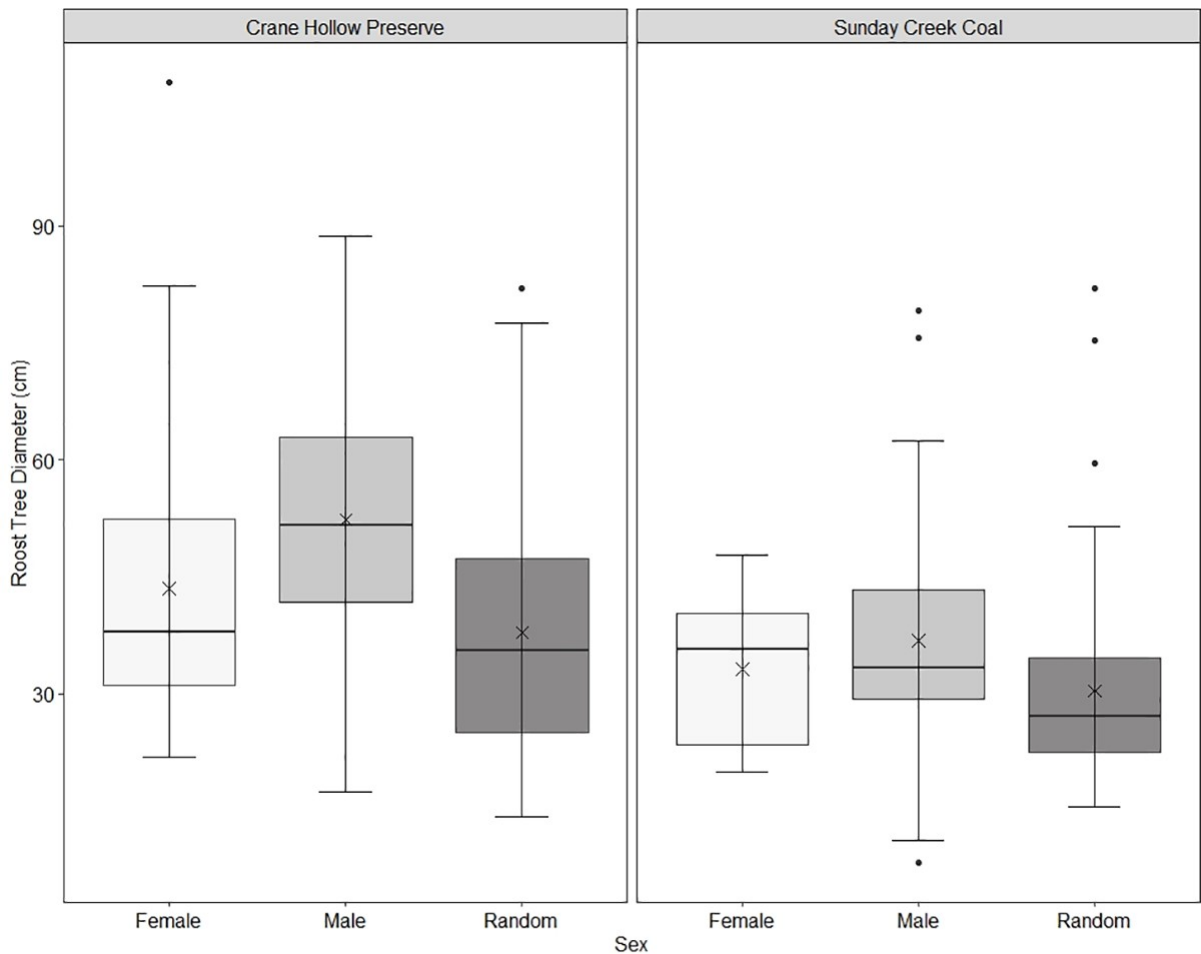
Box and whisker plot showing roost tree diameter of male (light grey) and female (white) eastern red bats at Crane Hollow Preserve and Sunday Creek Coal study areas in southeast Ohio, 2016–2019. Males roosted in larger diameter trees while the diameter of female roosts was similar to that of randomly sampled trees. Medians are depicted by the solid line, means are represented by the X’s, and outliers are depicted as black dots. Quartiles 2 and 3 are represented by the box and quartiles 1 and 4 are represented by whiskers.

The best supported model from Crane Hollow Preserve was the Tree Diameter and Elevation Model, which received 69% of the overall model support (Table 2). No other model had ΔAICc ≤ 2 [40]. Odds ratios of variables in this model show that the variable with the largest impact on probability of a tree being used by females was elevation (odds ratio = 1.151, CI = 1.151–1.153), but less so for males (odds ratio = 0.986, CI = 0.98–0.99). These ratios show that for every meter increase in elevation, the likelihood that a bat would occupy a roost increased by 15% for females but decreased by 1% for males (Fig 1). Females roosted within a narrow range of elevations ($\bar{x}$ = 330 ± 0.07 m, range = 319–334 m) while males ($\bar{x}$ = 319 ± 0.41 m, range = 251–331) and random trees ($\bar{x}$ = 321 ± 1.46 m, range = 282–335) were found across a wider range (Fig 2). For males, the variable with the largest effect on probability of a tree being used was DBH (odds ratio = 1.05, CI = 1.027–1.083). This indicates that for every centimeter increase in diameter of a tree, the odds that a male will use that tree for a roosting location increases by 5% (Fig 1). However, diameter was not as informative as elevation in driving female selection (odds ratio = 1.02, CI = 1.002–1.05, Fig 1). Mean diameter of male ($\bar{x}$ = 52 ± 0.52 cm), female ($\bar{x}$ = 44 ± 0.30 cm), and random trees ($\bar{x}$ = 38 ± 1.55 cm) reflect these odds ratios (Fig 3).

**Table 2:** Top 4 models for day-roost selection by male and female eastern red bats, along with the number of parameters, AICc score, ΔAICc score, and model weight for each, for Crane Hollow Preserve.

Model	K	AICc	Δi	wi
Diameter + Elevation	4	334.15	0	0.686
Elevation + Stand	6	336.97	2.81	0.245
Tree Size Model 1 + Elevation	5	337.71	3.55	0.116
Elevation + Distance to Water	4	340.62	6.46	0.027

AIC scores <2 indicates top model.

In contrast to Crane Hollow Preserve, the best supported model for Sunday Creek Coal was the Elevation and Distance to Water Model, which received 72% of the overall model support (Table 3). No other model had ΔAICc ≤ 2. Odds ratios of variables in this model show that elevation was the variable with the largest impact on the probability of a tree being used by both males (odds ratio = 1.05, CI = 1.02–1.077) and females (odds ratio = 1.034, CI = 1.025–1.077). Thus, each meter increase in elevation had a lesser effect on the probability of a tree being used by females compared to Crane Hollow Preserve (3% versus 15% increase) but a greater effect for males (1% decrease versus 5% increase, Fig 1). As a result, males ($\bar{x}$ = 297 ± 0.72 m, range = 285–304) and females ($\bar{x}$ = 294 ± 0.59 m, range = 251–331) occupied similar ranges of elevations at Sunday Creek Coal. Random trees were found across a wider range of elevations ($\bar{x}$ = 283 ± 1.71 m, range = 242–319) (Fig 2). Distance to water was an explanatory variable in the model for both males (odds ratio = 1.005, CI = 1.0002–1.0009) and females (odds ratio = 1.009, CI = 1.003–1.015, Fig 1), with both sexes roosting farther from water sources (females: $\bar{x}$ = 204 ± 3.22 m, males: $\bar{x}$ = 164 ± 3.66 m) compared to random trees ($\bar{x}$ = 120 ± 9.40 m).

**Table 3:** Top 4 models for day-roost selection by male and female eastern red bats, along with the number of parameters, AICc score, ΔAICc score, and model weight for each, for Sunday Creek Coal.

Model	K	AICc	Δi	wi
Elevation + Distance to Water	4	242.04	0	0.720
Tree Size Model 1 + Elevation	5	245.75	3.71	0.113
Slope Aspect + Elevation	5	246.67	4.64	0.070
DBH + Elevation	4	248.07	6.03	0.035

AIC scores <2 indicates the top model.

### Ambient temperatures

**Fig 4:**
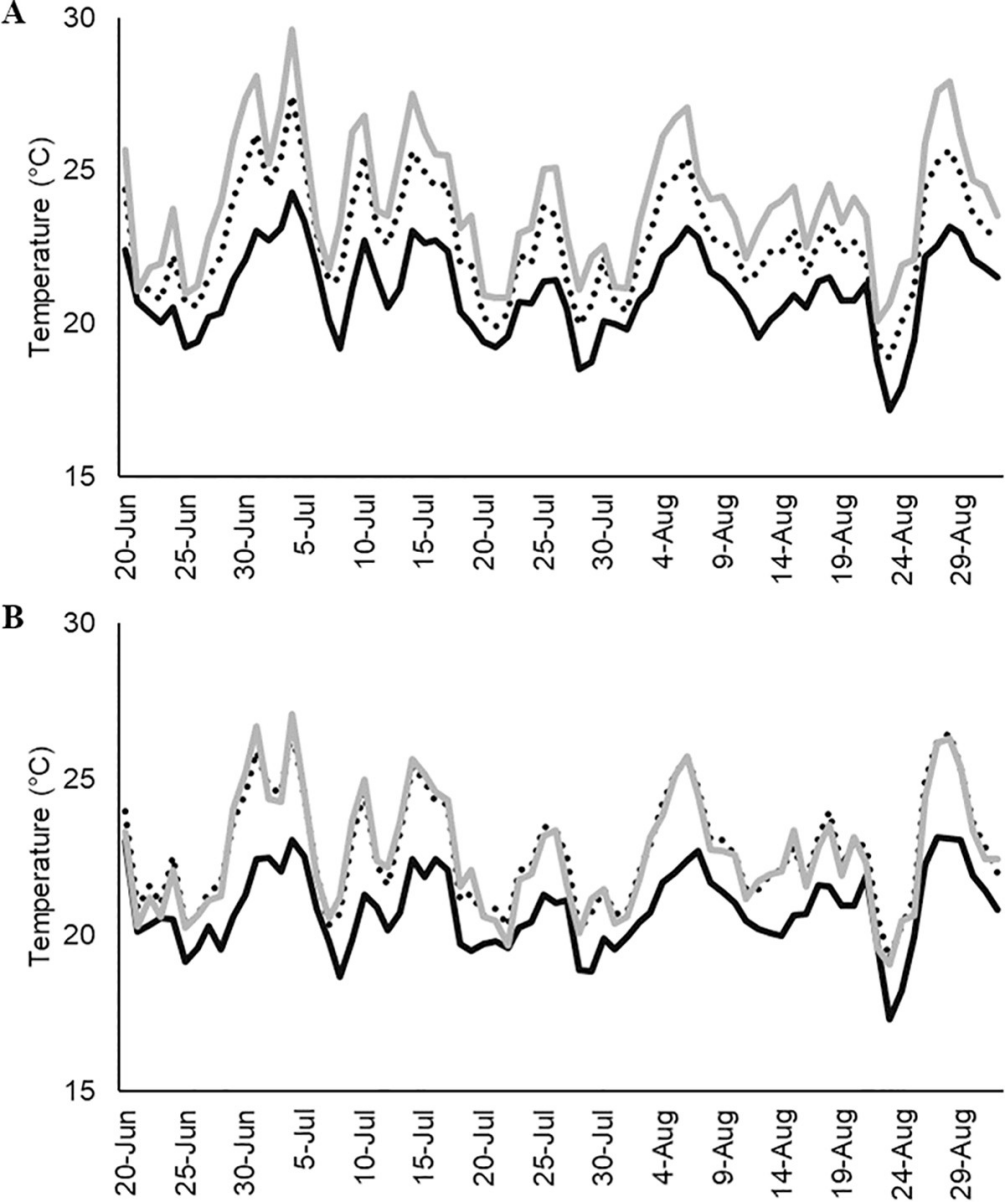
Average daily ambient temperatures were consistently warmer at the highest local elevations present at our two study areas, Crane Hollow Preserve (panel A) and Sunday Creek Coal (panel B) southeast Ohio from 20 June–29 August 2018. The dark, dashed, and gray lines represent temperatures collected from the bottom, middle, and upper elevations sampled.

Ambient temperatures varied across the elevational gradient at both sites. Ridgetops had slightly warmer average daytime temperatures from June–August at Crane Hollow Preserve ($\bar{x}$ = 24.0 ± 0.25° C) and Sunday Creek Coal ($\bar{x}$ = 22.6 ± 0.22° C) compared to lower elevations ($\bar{x}$ = 21.0 ± 0.16° C and $\bar{x}$ = 20.8 C ± 0.15° C, respectively). Similar elevations between the two sites varied slightly in temperature with Crane Hollow Preserve being slightly warmer on average (Fig 4).

## Discussion

We found that daytime habitat selection by eastern red bats in southeast Ohio varied between sexes and between study areas with different forest conditions. In a forest characterized by larger, less densely stocked trees, males and females selected day-roosts differently based on tree diameter and elevation. Female use was more affected by elevation while male use was more affected by tree diameter. Female roosts were located at higher elevations and in smaller diameter trees than males. However, in a forest with a similar elevational gradient characterized by a higher density of smaller trees, tree diameter was not important in roost selection, and male and female roosts overlapped more in elevation. These data show that patterns of habitat use by bats can vary between areas located in the same part of a species’ range, likely in response to forest structure and microclimate. This highlights the need to study habitat use across a range of environmental conditions to untangle local effects on habitat use.

The possibility that heterogeneity in environmental conditions can lead to differing effect sizes, and even directions, for variables influencing habitat selection in bats was recently explored by Fabianek and colleagues [41]. Focusing solely on cavity and bark roosting species, the authors found both consistent and inconsistent trends for the influence of variables measured in the present study, and concluded that summer temperatures moderate some, but not all, of this variability. The importance of temperature for small, insectivorous bats is well-known, as these small mammals lose heat quickly, and reproductive females benefit from selecting day-roosts that minimize this heat loss [23, 42]. Warmer maternity roost temperatures are also essential for juvenile development, as offspring develop quicker under warmer maternity roosting conditions [43]. Due to the ongoing pressure to be ready for migration and subsequent hibernation, fast development is important for juvenile survivorship [44, 45]. Therefore, a frequently appearing narrative is that reproductive females select roost in trees located at lower elevations where conditions are warmer [40, 46, 47]. Our results point to different effect of elevation, that eastern red bats, especially reproductive females, roost at higher elevations, but with the same inference: that female bats often chose to roost in warmer areas on the landscape. Unlike the studies cited above, lower elevations were associated with lower temperatures in our study area. This difference in the thermal gradient likely stems from the scale of topography between our sites in southeastern Ohio, where elevation only varied by approximately 100 m, often in the form of narrow, steep gorges. Despite the subtle difference in elevation in our study, temperatures differed by ~3° C from ridges to bottoms, a biologically meaningful difference from a thermoregulatory perspective.

It is notable that females at Crane Hollow Preserve were more likely to select roosts at higher elevations in comparison to males. Sexual segregation in bats has been observed by others, in which, females occupied lower, warmer elevations, than males during pregnancy and lactation [40, 46, 47]. Males are less energetically constrained than females, and likely experience fewer costs associated with roosting at lower temperatures [17, 48]. However, lesser energetic constraints in males does not fully explain why males at Crane Hollow Preserve roosted at a different range of elevations than females, as roosting at colder elevations would increase the cost of maintaining normothermy for males. This finding suggests either that lower elevations provided males with a benefit such as the ability to use deeper torpor or the opportunity to avoid competition with bats in higher quality (warmer) microclimates.

Meta-analyses and reviews of day-roost selection consistently find that tree diameter is a primary predictor of whether a tree is used by bats [29, 41]. Larger diameter trees are thought to benefit bark and cavity roosting species because these trees provide more room for roosting groups of bats [16, 22]. Larger trees have also been hypothesized to have thicker bark and are sometimes located in more open areas, making the tree a better thermal environment for roosting [49]. These explanations are unlikely to explain why red bats preferred larger diameter trees at the preserve, as increased space for social roosting would not benefit solitary red bats, and roost trees were not located in open habitats. It is also difficult to explain why males would roost in larger trees than females. Tree diameter at this site was not correlated with elevation, and we saw no evidence that larger trees were available at lower elevations, which could have otherwise helped to explain this difference. Thus, while it is apparent that several aspects of larger trees can benefit other bat species, it’s unclear why foliage roosting species would prefer larger diameter trees.

Although our results do not provide insight into why tree diameter is important for red bats, it is clear from our results that sexes selected roosts differently when larger diameter trees were available. However, at Sunday Creek Coal, where we caught fewer red bats overall and females were rare, we found no evidence of sexual segregation. We hypothesize that this difference was the result of forest conditions in each area. We propose that dense forests of relatively small diameter trees, such as that at Sunday Creek Coal, are marginal roosting habitats for eastern red bats. In such areas, males and females either fail to segregate where they otherwise would, or female populations are so low as to eliminate the need for segregation, although further studies are needed in this area. Regardless, our results provide clear support of previous studies showing that forest structure is an important aspect of day-roosting habitat for the eastern red bat in southeastern Ohio [26, 29, 31].

Finally, distance to water was a variable in our top model at Sunday Creek Coal. Proximity to water is important for many bats, which often drink and forage in the area surrounding their roosts upon evening emergence [15, 50]. However, we observed the opposite trend, with both male and female eastern red bats roosting farther from permanent water sources than available trees. Because it is unlikely that bats seek to roost far from water, it is likely that this metric either failed to capture water availability or was correlated with another, unmeasured variable. Ephemeral water sources regularly used by bats, such as puddles in forest roads or vernal pools, were found at higher elevations but were not be accounted for in our study. Regardless, the average distance measured between the water sources and roosts was 200 m, a distance easily traversed by bats, and unlikely to impact them negatively [51].

The underlying motivation for many studies of bat habitat selection is to guide management practices. Over the last several decades, a large body of work has emerged describing the habitat of different species in different regions, with some clear trends emerging [2, 29, 41]. Unfortunately, many of these trends are aggregated across species and are biased towards studies of cavity- and bark-roosting species. However, foliage roosting species are also experiencing significant population declines, and more research is needed to guide management for these species as well. While our netting data cannot be used to compare relative abundance of each sex in our study areas, it is notable that we only captured 7 female red bats at Sunday Creek Coal over four years of effort. It is therefore likely that females are rare in this area. Regional based differences in sex ratios are common for bat species and have been well-documented in eastern red bats. Although it is not uncommon for surveys to document these male-biased sex ratios, data are often insufficient to make clear conclusions why [40, 46, 47]. Temperature as a function of elevation [40, 46, 47], resource distribution [46], and seasonal patterns [52] have often been the primary hypotheses explaining these differences. However, given the close proximities of our study locations and the similarities in temperature regimes, habitat quality may be another variable to consider when parsing out what is driving some of these patterns. We encourage researchers to study habitat use within different forest contexts, beyond different silvicultural treatments, and over different temperature regimes. Investigations of where female reproduction does and does not occur in reference to these characteristics across a species’ range can lead to better understanding of factors necessary for reproduction, driving habitat use, and helping guide management and conservation efforts.

In the absence of these studies, our work highlights the importance of maintaining diversity in forest structure over elevational gradients for eastern red bats in southeastern Ohio. We suggest retaining trees > 38 cm DBH (based on our overall average roost diameter at Crane Hollow Preserve) across an elevational gradient to provide suitable roost trees for eastern red bats. To improve habitat in forests such as Sunday Creek Coal, single tree selective thinning or group selection thinning could be employed to open the understory and release remaining trees from competition. Despite our data suggesting that sugar maples were the only tree species used more than expected by females, we also recommended retaining tulip poplars, oaks, and sweet birch since these species were frequently used at both study sites, indicating they are suitable roosts. While the specific effects of the variables we report must be viewed in the context of our study areas where the topography created cooler temperatures in riparian areas and at the bottom of gorges, the inference that temperature has a strong influence on habitat selection by eastern red bats may broadly apply throughout the species’ range. We urge land managers to provide roosting habitat in relatively warm microclimates where thermal conditions vary on the local conditions.

## Supporting information

S1 Table Biologically informed a priori models and corresponding variables used for our information theoretic approach and multinomial logistic regressions. Superscripts provide citations justifying the variables in each model.
(PNG)
