# Supplementary figures and images for: Summer day-roost selection by eastern red bats varies between areas with different land-use histories
Source: PLoS One. 2020 Aug 24;15(8):e0237103. doi: 10.1371/journal.pone.0237103 (PMC7444818; doi:10.1371/journal.pone.0237103)

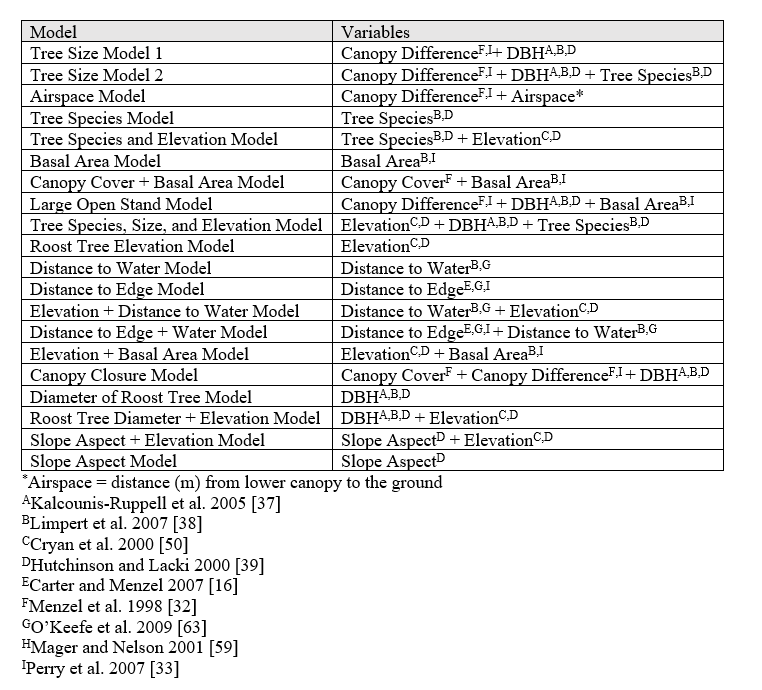

Supplement: S1 Table — Superscripts provide citations justifying the variables in each model. (PNG) [file pone.0237103.s001.png]
